# The Art of the Editorial

**DOI:** 10.3126/nje.v12i1.43104

**Published:** 2022-03-31

**Authors:** Edwin R. van Teijlingen, Vanora Hundley, Brijesh Sathian, Padam Simkhada, Jared Robinson, Indrajit Banerjee

**Affiliations:** 1,2 Centre for Midwifery, Maternal and Perinatal Health, Bournemouth University, Bournemouth, United Kingdom; 3 Geriatrics and long term care Department, Rumailah Hospital, Hamad Medical Corporation, Doha, Qatar; 4 School of Human and Health Sciences, University of Huddersfield, United Kingdom; 5, 6 Sir Seewoosagur Ramgoolam Medical College, Belle Rive, Mauritius

## Background

Traditionally an editorial offered the readers a unique insight into perspective of the editor writing the editorial to highlight the connections between the papers included in the latest issue of the journal, perhaps linking it to key scientific, policy or political events in the journal’s field. Sometimes the editorial focuses on just one important paper within the journal. Editorials may also focus on hot topics in the field offering the views and perceptions of an expert writing the editorial on a topic of interest. Occasionally journals decide on a Special Issue on a particular topic. This Special Issue may have a guest editor who also writes the accompanying editorial highlighting the theme or themes that link the articles in the Special Issue.

Unfortunately writing editorials is becoming a little bit of a lost art. Nearly a decade ago Ruckdeschel and Shaw wrote that there has been “a trend for some journals to skip editorials and to simply publish the article in the order accepted” [1]. Today there are more academic journals on the market that do not carry editorials and many of the more recently established online journals have done away with editorials. Possible explanations for this decline are that online journals don’t publish proper issues at a regular interval, but instead they published each paper as it is accepted as they want to get the information out as soon as possible. We feel that is a pity as editorials can be interesting commentaries and they have an important place in the world of scientific publishing.

Over the past two decades we have published close to fifty editorials between us. We would like to highlight some of their features to (a) offer advice to would-be editorial writers, (b) encourage more junior academic to embrace the writing of editorial as a scholarly activity, and at a grander level (c) help prevent the demise of the editorial.

### The role of editorials

Editorials help journals to distinguish themselves; a journal that features an editorial is not just a collection of papers but has something personal to say to its readers, which may come from the editor (origin of the term) or an invited author. For example, the editors of the Journal of Asian Midwives often stress the importance of the ICM (International Confederation of Midwives) in their editorials [2-4]. The content of the editorial helps put the editors’ personal stamp on the identity of their journal [5]. The editorial allows editors to communicate with their readers and their (potential) contributors [5]. An obvious example of this would a call for papers for a Special Issue of the journal [6]. Editorials can help highlight certain papers in the current issue of the journal which the editor thinks are outstanding or particularly innovative. Peh WC et al. highlight five purposes of an editorial that reflect these aspects of communication, see Box 1: [5]

**Box 1: Purposes of an editorial:**

- Personal message from the editor to journal readers
- Commentary on published article in the same issue
- Concise review on a topic of current topic (not warranting a full invited review)
- Drawing readers’ attention to very recent development or innovations
- Commentary on non-scientific topics. E,g, health policy, economics, law or ethics

However, we feel that an editorial frequently go beyond simple communication, and if well written will stimulate thinking and scientific discourse. As Singh and Singh challenge, a good editorial is not always comfortable to read, but it does set out to make the reader think [7]. Sometimes editorials have an education element, for example, on aspects of academic publishing [8-9], or the value of qualitative research [10]. Editorials can also comment on political, social, cultural or economic events, hot topics or technical or legal developments that are particularly relevant to the academic discipline represented by the journal [5]. For example, two of us got together with various midwifery colleagues to write about the Millennium Development Goals coming to an end in 2015 [11] and about mental health policies in Nepal [12]. More recently several of us wrote about the COVD-19 pandemic when it first occurred [13-16]. As Singh and Singh reminded us: “Scratch the surface of any good editor who enjoys his job, and a crusader will shine through” [7]. Editorials can also be opinion pieces challenging established views and pushing out the boundaries of knowledge.

Editorials can serve many other purposes, “including critiques of original articles published in the same issue of the journal, concise reviews of topics that do not warrant a full-length invited review, and other topics on very recent developments that are deemed by the editor to be important to readers of the journal and the community” [5].

### Who writes editorial?

Journal editors edit and direct journals and as part of this role they sometimes write editorials. They are usually unpaid volunteers who act as editors over and above their day job [17]. The latter results in editors often inviting experts to write a so-called guest editorial. For example, on the publication of an important report the ground-breaking Global State of the World’s Midwifery Report (SoWMy) in 2011, one of us was invited to write a guest editorial for the International Journal of Childbirth [18].

### How to write an editorial

It is a good idea to keep editorials brief and to the point, highlighting one single issue. The online journal Social Inclusion (https://www.cogitatiopress.com/socialinclusion/pages/view/forauthors) advises would-be editorial writers: “Editorial: an introductory piece submitted by an Academic Editor providing insight into the topic of the thematic issue. Editorials shall have a maximum length of 2,000 words (the word count limit includes title, abstract, tables, figures, and references list).” Interestingly, Social Inclusion has a publication charge for standard paper, but: editorials and commentaries up to 2,000 words are free of charge.

Editorials must be written in clear but engaging language; as Gray said “A good editorial should have a quinoa effect. Love or loathe the stuff it gets a reaction” [19]. However, there is a need for caution as too much polemic will have the opposite effect. Leslie and Hemmings adopt the acronym GRACE as a guide for writing editorials [20] (see Box 2).

**Box 2: GRACE guide for writing editorials [20]**

**G**racious: discuss strength and limitations respectfully.

**R**elevant: provide opinions relevant to the paper and its readers.

**A**ccurate: quote the paper and literature accurately and conform to journal requirements.

**C**ontroversial: express and opinion and advance the discussion in a thoughtful and provocative way.

**E**ngaging: write for a general readership, medical journalists, social media, and the public.

### Insights from Nepal Journal of Epidemiology during the COVID era

All eight of the last editorials published in the Nepal Journal of Epidemiology have focused on the SARS-CoV-2 virus and its impact on not only Nepal, but the global community as a whole. These editorials aimed to bring readers the latest information as new aspects of the novel virus came to light. The editorials developed along with the latest scientific information, thereby conveying that the editors had their fingers on the proverbial “pulse” of the COVID-19 pandemic. The natural evolution of the editorials is evident as the initial editorials focused on the early stages of the pandemic and the emerging epidemiology as well as highlighting areas of weakness which needed strengthening within the context of COVID-19 and Nepal [13]. Shortly after the COVID-19 pandemic got a foothold into the global community national lockdowns and self-isolation were the mainstay method used in combatting the spread of the virus. The rapid change in society from highly social to complete self-isolation coupled with the fear of the unknown of this novel virus caused unprecedented psychological stress within the community. The psychological impact thereof was highlighted through the second editorial published in the COVID era [13]. The following editorials published by the journal homed in on methods in which the virus and spread thereof could be best curtailed and minimized. The two following editorials focused on prohibition of alcohol and artificial intelligence as methods which could be used to best combat and minimize the effect of the virus [14, 21]. The Nepal Journal of Epidemiology, thus provided information on useful and practical methods being employed by other countries to reduce transmission of the virus and thus offered readers an array of options which could best be applied in their respective country [14, 21]. The journal subsequently and in character with bringing readers the latest from the global scientific community published an editorial on the mutations of the SARS-CoV-2 virus as well as addressed the fear and question of “reinfections”. It was concluded that reinfections were lower and less severe in individuals who had been vaccinated [22]. In pandemics chronic medical conditions such as cancer get neglected due to fears of undergoing surgeries or approaching healthcare facilities in fear of contracting the virus. The sixth COVID-19 based editorial highlighted this issue and substantiated the safety of undergoing such robotic and contactless surgery during the pandemic [23]. The rigorous treatment protocol for patients suffering from COVID-19 called for the use of high doses of corticosteroids. The long-term implications thereof as well dosing to prevent avascular necrosis was critically discussed in the editorial and thus again highlights the practical sense of the editorials published by the Nepal Journal of Epidemiology in the Covid-19 series [24]. The most recent editorial published by the journal explores the alarming rise of suicides in Nepal which is attributed to the stressors induced by COVID-19 as well as the methods in which such events can be prevented [25]. The eight editorials published thus far have been both informative and applied aiming to inform readers about the most up-to-date insights and information available in the pandemic. The natural evolution of the editorials and their topics stand as a testimony to the Nepal Journal of Epidemiology and their level of quality and drive to keep readers informed with the most practical and interesting information available.

### Final thoughts

We have taken ideas from Leslie and Hemmings [20], Gray [19] and our own experiences and combined these into the guidance listed in Box 3. As Leslie and Hemmings commented: An editorial “gives the writer a podium on which to flex their scientific and literary muscle, stimulate scientific discourse, and influence their colleagues for the greater good.” [20]. We would like to go one step further than Leslie and Hemmings, we would argue that writing an editorial not only offers the academic a platform, it also can be steppingstone for their career. To finish this editorial, we conclude that being invited to contribute an editorial for a good scientific journal is a great honour.

**Box 3: Basic advice for writing an editorial for an academic journal**

- Keep the editorial short and clear.
- Keep it topical.
- Follow the journal’s author instructions.
- Follow the brief (if you are invited by the editor).
- Have one clear slightly challenging or controversial argument.
- Balance your controversial argument by acknowledging our views and perspectives.
- Don’t make it an essay with too much detail.
- Write for your audience, ask yourself: ‘Who reads the journal?’

## References

[1] Ruckdeschel R Shaw I. Reflections on editing a qualitative journal, Qualitative Social Work. 2013;12(6):750-764. https://doi.org/10.1177/1473325013488619 10.1177/1473325013488619

[2] Jan R van Teijlingen E. Developments in midwifery in Asia in 2017. J Asian Midwives. 2017;4(2):1-2.

[3] Jan R van Teijlingen E. Asian Midwifery in 2018. J Asian Midwives. 2018;5(1):1-3.

[4] Jan R van Teijlingen E. Midwifery 2021 in a World in Turmoil. J Asian Midwives, 2015;8(1):1-2.

[5] Peh WC Ng KH. Writing an editorial. Singapore Med J. 2010 Aug;51(8):612-5. PMID: . 20848056

[6] van Teijlingen E Homer C Sandall J. Special issue of Midwifery on ‘The maternity work force’: Call for papers. Midwifery 2010;26: 257. https://doi.org/10.1016/j.midw.2010.03.008 10.1016/j.midw.2010.03.008

[7] Singh A Singh S. What is a good editorial? Mens Sana Monogr. 2006 Jan;4(1):14-7. https://doi.org/10.4103/0973-1229.27600 10.4103/0973-1229.27600 PMid: PMCid: 22013327 PMC3190447

[8] Hundley V van Teijlingen E Simkhada P. Academic authorship: who, why and in what order? Health Renaissance 2013;11(2): 98-101 https://doi.org/10.3126/hren.v11i2.8214 10.3126/hren.v11i2.8214

[9] Simkhada P van Teijlingen E Hundley V. Writing an academic paper for publication, Health Renaissance 2013;11(1): 1-5. https://doi.org/10.3126/hren.v11i1.7592 10.3126/hren.v11i1.7592

[10] Simkhada P van Teijlingen E Wasti SP Sathian B. Mixed-methods approaches in health research in Nepal. Nepal J Epidemiol 2014;4(5): 415-416. https://doi.org/10.3126/nje.v4i5.11993 10.3126/nje.v4i5.11993 PMC9057171 35528457

[11] van Teijlingen E Hundley V Matthews Z. Millennium Development Goals: All good things must come to an end, so what next? Midwifery 2014; 30: 1-2. https://doi.org/10.1016/j.midw.2013.10.022 10.1016/j.midw.2013.10.022 PMid: 24238981

[12] Simkhada PP van Teijlingen E Marahatta SB. Mental health services in Nepal: Is it too late? J Manmohan Mem Inst Health Sci 2015;1(4): 1-2. https://doi.org/10.3126/jmmihs.v1i4.12012 10.3126/jmmihs.v1i4.12012

[13] Asim M van Teijlingen E Sathian B. Coronavirus Disease (COVID-19) and the risk of Post-Traumatic Stress Disorder: A mental health concern in Nepal. Nepal J Epidemiol. 2020 Jun 30;10(2):841-844. https://doi.org/10.3126/nje.v10i2.29761 10.3126/nje.v10i2.29761 PMid: PMCid: 32874697 PMC7423405

[14] Banerjee I Robinson J Sathian B van Teijlingen ER. South Africa and its COVID-19 prohibition predilection. Nepal J Epidemiol. 2020 Sep 30;10(3):874-877. https://doi.org/10.3126/nje.v10i3.31543 10.3126/nje.v10i3.31543 PMid: PMCid: 33042590 PMC7538013

[15] Jan R van Teijlingen E. COVID-19: The New Corona Virus Upsetting Our World. J Asian Midwives 2020;7(1):1-3.

[16] van Teijlingen E Sathian B Simkhada P Banerjee I. COVID-19 in Qatar: Ways forward in public health and treatment. Qatar Med J. 2021 Jan 4;2020(3):38. https://doi.org/10.5339/qmj.2020.38 10.5339/qmj.2020.38 PMid: PMCid: 33447537 PMC7780299

[17] van Teijlingen E Thapa D Marahatta SB Sapkota JL Regmi PR Sathian B. Editors and reviewers: Roles and responsibilities, In: Wasti. (Eds.) Academic Writing and Publishing in Health and Social Sciences (Chapter 5). Kathmandu, Nepal: Social Science Baha & Himal Books, 2022.

[18] van Teijlingen E. The State of the World’s Midwifery (Editorial) Int J Childbirth 2012;(4): 214-5.

[19] Gray R. Now hang on a minute: five rules for writing an editorial. J Psychiatr Mental Health Nurs. 2015; 22: 559-560. https://doi.org/10.1111/jpm.12260 10.1111/jpm.12260 PMid: 26337593

[20] Leslie K Hemmings HC Jr. Excellence in editorials: fulfilling their critical role in medical literature. Br J Anaesthesia 2020;125(5): 639-641. https://doi.org/10.1016/j.bja.2020.06.061 10.1016/j.bja.2020.06.061 PMid: 32828491

[21] Banerjee I Robinson J Kashyap A Mohabeer P Sathian B. COVID-19 and Artificial Intelligence: the pandemic pacifier. Nepal J Epidemiol. 2020 Dec;10(4):919. https://doi.org/10.3126/nje.v10i4.33334 10.3126/nje.v10i4.33334 PMid: PMCid: 33495709 PMC7812328

[22] Banerjee I Robinson J Sathian B. COVID-19: Are reinfections a global health threat? Nepal J Epidemiol. 2021 Mar;11(1):933. https://doi.org/10.3126/nje.v11i1.34903 10.3126/nje.v11i1.34903 PMid: PMCid: 33868739 PMC8033645

[23] Banerjee I Banerjee I Banerjee S. Is Robotics the real game changer for Urological cancer care during COVID-19 crisis? Nepal J Epidemiol. 2021 Jun;11(2):988. https://doi.org/10.3126/nje.v11i2.38133 10.3126/nje.v11i2.38133 PMid: PMCid: 34290889 PMC8266403

[24] Banerjee I Robinson J Sathian B. Corticosteroid induced avascular necrosis and COVID-19: The drug dilemma. Nepal J Epidemiol. 2021 Sep;11(3):1049. https://doi.org/10.3126/nje.v11i3.39309 10.3126/nje.v11i3.39309 PMid: PMCid: 34733566 PMC8560140

[25] Banerjee I Robinson J Sathian B Banerjee I. COVID-19 pandemic and suicides in Nepal: Way forward for prevention. Nepal J Epidemiol. 2021 Dec 31;11(4):1083-5. https://doi.org/10.3126/nje.v11i4.41116 10.3126/nje.v11i4.41116 PMid: PMCid: 35070467 PMC8730343

